# Collaborative Research on Mouth Shape and Lyrics in Singing Practice Based on Image Processing

**DOI:** 10.1155/2022/5138442

**Published:** 2022-01-28

**Authors:** Lujia Xu, Chen Chen

**Affiliations:** ^1^Xiamen University Tan Kah Kee College, Zhangzhou 363123, China; ^2^The First Affiliated Hospital of Xiamen University, Xiamen 361000, China

## Abstract

Image processing is a mainstream processing method. When people enjoy artists' singing videos, there will be a problem that the subtitles of the lyrics are out of sync with the singer's mouth shape. This problem needs to be solved using image processing technology, letting the computer realize lip-reading recognition function and correct the mouth shape and lyrics subtitles in the image according to the extracted lip-reading data, so that the mouth shape and lyrics in singing practice can be synchronized. Lip-reading information can effectively improve the accuracy of language cognition, save part of capital and manpower investment, and make viewers get a good audio-visual interactive experience. The results show the following: (1) After the UI test, the system user interface function design is reasonable and there is no bad BUG. We can find that the average processing time of each frame is 628 ms, the system performance evaluation is good, and the success rate can be as high as 98.80%. 0.36724 s is the average time for each step when the system processes the image. (2) The human image can basically identify the portrait area and lip area from various angles. (3) Compared with DCT and DWT, the recognition rate of the two cascade lip region feature extraction methods is improved by nearly 10%, and the feature vector dimension is reduced by nearly 65%. (4) Classify the mouth shape more finely and optimize the image of the tester's mouth shape to make the mouth shape closer to the standard mouth shape. (5) After systematic correction of mouth shape and subtitles, the success rate is higher than 90%. Finally, we can find that the running effect is good and the method has achieved high results, which can carry out the details of the next optimization work.

## 1. Introduction

With the spring breeze of science and technology sweeping the world, times have quietly changed dramatically. How to use the power of science and technology to make people's daily life more efficient and convenient is the most frequently considered problem nowadays. In recent years, people are using lip-reading more and more frequently, although they do not realize that they are using it. However, lip reading technology is no longer a “spring snow.” It has gone out of the laboratory and gradually stabilized from little known to development and research. The relevant application technology is becoming more and more practical, and more and more people from outside are beginning to understand and learn its technology and characteristics. In this study, if we want to coordinate the mouth shape and lyrics in the video, the first step is to accurately locate people's lips in the image, and then a series of mouth shape recognition tasks are carried out. There are many literature and materials related to computer lip-reading recognition in existing journals, papers, and other places, and we select some of them for reference study here. Literature [1] introduces the reality and development level of lip reading, which arouses people's attention and interest. Literature [2] proposes algorithms for lip detection, feature extraction, and other technologies to realize a lip-reading prototype system. In [3], the face recognition technology is used in the attendance system. Literature [4] uses optical flow to analyze lip images, calculates two visual feature sets in each frame, and proposes a multimode speech recognition method. Literature [5] analyzes lip-reading technology according to typical deep learning and lists the existing lip-reading databases. Literature [6] uses a three-dimensional motion capture system to extract accurate parameters of facial motion features through lip reading. Literature [7] describes the public database of lip reading, target speech content, equipment, camera orientation, frame rate, and application. In [8], the coupled hidden Markov model is used to combine audio-visual signals with Polish speech recognition under the condition of highly interfered audio signals. Literature [9] studies the dual-mode fusion algorithm of video and audio, combining lip-reading speech recognition and information fusion technology. Literature [10] tests the fusion technology of different modes and analyzes different methods of audio-visual speech recognition. Literature [11] proposed the application of a lip-reading method based on a convolution neural network to tandem three-sequence key frame images. Literature [12] developed the deep convolution neural network model of HLR-Net for lip reading. Literature [13] proposes a new VSR method, multiangle lip reading, for audio-visual speech recognition. Literature [14] classifies those binoculars, enzymes, and muscle contractions in language when lip reading using multichannel SEMG signals. Literature [15] designs a graphic structure and lip partial cut network, using a local adjacent function extractor and lip reading with multilevel function fusion.

## 2. Theoretical Basis

### 2.1. Video and Audio Bimodal Corpus

Video and audio bimodal corpus [16] serves as the research basis. It will process the video according to the speech signal and visually include various face location images and mouth images related to the speech pronunciation. Because corpora involve many factors that need to be considered, such as format, coding, environment, and storage, and because there are few bimodal corpora that can be shared in the market, there are Tulips, M2VTS, ViaVoice, and other corpus databases in the world. There are the relevant parameters of the corpus, as shown in Table 1.

### 2.2. Lip Detection and Positioning Method

Different lip feature extraction methods obtain different lip areas [17]. If the first step of this positioning is not successful, all the functions based on lips will not be realized smoothly. The detection of lip area requires the system to quickly and accurately find the approximate range of the face in each frame of an image. After face recognition, it is necessary to accurately locate the lips according to their characteristics. In this way, we can ensure that after the previous work, we have laid a good foundation for lip feature extraction and make the subsequent image processing smoother and easier. The following is an introduction to typical lip detection methods.

#### 2.2.1. Face Detection

Observing the facial features of human beings, there are three main human organs: eyes, nose, and mouth. Unless there are man-made or unexpected factors, their positions will not change much for a long time, and they have relative stability. Based on the stable features of face organs, we can use computer programs to detect the specific location of the face, and then use the Canny operator and projection method to quickly locate the lip area. *W*_f_ denotes the face width and *H*_f_ denotes the face height and the lip area of interest is as follows:

(1) $$\begin{array}{c} \frac{1}{4}W_{f}\leq x\leq \frac{3}{4}W_{f},\\ \frac{2}{3}H_{f}\leq y\leq \frac{1}{15}H_{f}. \end{array}$$

However, due to the limitations of technology, time, and space, this detection method cannot accurately obtain and recognize the position of the face and specific lip area in some cases. This is because, in the process of singing, different singers have different faces, hairstyles, and postures. If there is a certain movement range (even sometimes there will be intense dance movements) to perform, it is impossible for the face to keep a positive and stable posture for detection at any time, and the detection efficiency is greatly reduced. Moreover, the relative position and size of the lip area will change with factors such as speech and expression. Therefore, this method is only suitable for preliminary and rough determination of lip area, which reduces some calculation work, but further accurate detection and confirmation are needed.

#### 2.2.2. Color-Based Detection

According to the color distribution of the human face, we can find that the color of lips of most people is quite different from that of other areas of their face. Therefore, we can analyze the color intensity and color interval of lips according to different colors so as to correctly detect the parts of lips.

Because the red part of the face and lips account for different proportions. We usually choose to exclude the red gamut in the face by the red exclusion method, which is very classic and can effectively promote the solution of the problem. After reducing the influence of the red part on color detection, the situation represented by the original red area is replaced by blue and green. Here, we use the concept and meaning of three primary colors (RGB). This method can save the steps of color space conversion and tedious workload. There is also a method called the pseudocolor method, which is used to enhance the contrast between skin color and lip color. Finally, the image is converted to YIQ space, excluding the Y component, and good color discrimination can be seen. The formula is as follows:

(2) $$\begin{array}{c} \log\left(\frac{G}{B}\right)<\beta , \end{array}$$

(3) $$\begin{array}{c} \left[\begin{array}{c} Y\\ I\\ Q \end{array}\right]=\left[\begin{array}{ccc} 0.299 & 0.587 & 0.114\\ 0.596 & -0.275 & -0.321\\ 0.212 & -0.528 & 0.311 \end{array}\right]\left[\begin{array}{c} R\\ G\\ B \end{array}\right]. \end{array}$$

Formula (2) is a discriminant, where *G* and B represent green and blue component values, respectively, which are thresholds calculated by statistics. If the discriminant is satisfied, the pixel becomes a lip color pixel, otherwise it becomes a skin color pixel. Formula (3) is the formula of color intermediate conversion, which converts the image from RGB space to YIQ space, where Y is the gray component and I and *Q* are the chrominance components.

However, it should be noted that people have different skin tanning degrees, and each person's lips and skin color are different, so the specific colors need to be classified and discussed differently, which is not universal and brings more troubles to the specific recognition work. In addition, because singers will wear certain makeup because of social etiquette, work, and other factors, all kinds of lip gloss and cosmetics will change the color of their face, which greatly reduces the accuracy based on color detection.

#### 2.2.3. Model-Based Detection

This method mainly uses the key points above the lip shape in the image to confirm the contour of the lip. Using the ACM algorithm, we can quickly build a matching lip model. The calculation degree of this method is very complex. If it is applied in practice, the burden of image processing will be very large, and it is difficult to correct mistakes in time once they go wrong. The relevant formula is as follows:

(4) $$\begin{array}{c} E_{\text{Snake}}\left(V\right)=\overset{N}{\underset{i=1}{\sum }}E_{\text{Snake}}\left(i\right)=\overset{N}{\underset{i=1}{\sum }}\left(E_{\mathrm{int}}\left(i\right)+E_{\text{ext}}\left(i\right)\right),\\ E_{\mathrm{int}}\left(i\right)=\alpha_{i}{\left\|V_{i}-V_{i-1}\right\|}^{2}+\beta_{i}{\left\|V_{i-1}-2V_{i}+V_{i+1}\right\|}^{2},\\ E_{\text{ext}}\left(i\right)=E_{\text{image}}\left(i\right)+E_{\text{con}}\left(i\right). \end{array}$$

**Figure 1:**
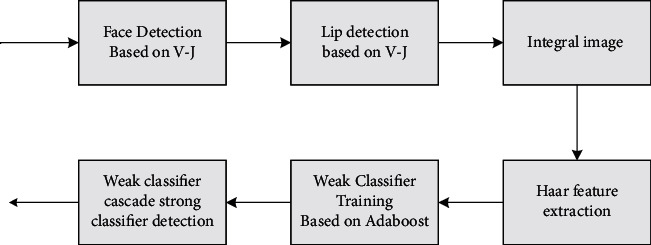
Viola–Jones lip detection flow chart. In the Haar rectangle feature.

A Viola–Jones method for lip region detection was established [18]. In this paper, we choose to fuse the abovementioned lip detection methods, and combine the face detection method with Viola–Jones method to learn from each other's strengths. A flow chart related to lip area detection is shown in Figure 1.


(5) $$\begin{array}{c} ii\left(x,y\right)=\sum_{x^{\prime }\leq x,y^{\prime }\leq y}i\left(x{}^{\prime },y{}^{\prime }\right), \end{array}$$

(6) $$\begin{array}{c} s\left(x,y\right)=s\left(x,y-1\right)+i\left(x,y\right), \end{array}$$

(7) $$\begin{array}{c} ii\left(x,y\right)=ii\left(x-1,y\right)+s\left(s,y\right). \end{array}$$

In Figure 1, the Haar feature extraction module is mainly used. The value of integral image *ii* of any image *i* at any pixel *(x, y)* is defined as formula (8), and formula (9) and formula (10) can be obtained by calculation.

The training for AdaBoost is as follows:

(8) $$\begin{array}{c} h_{i}\left(x\right)=\left\{\begin{array}{cc} 1, & \sum_{t=1}^{T}a_{t}h_{t}\left(x\right)\geq \frac{1}{2}\sum_{t=1}^{T}a_{t},\\ 0, & \text{otherwise.} \end{array}\right. \end{array}$$

### 2.3. Image Processing Techniques

The digital image is represented by a two-dimensional array [19, 20]. Image processing technology has made more and more applications in recent years [21, 22]. Each pixel is unique, with unique plane position coordinates and values, and is the basic storage unit of digital images. In order to improve the clarity of the image, we can sharpen and enhance the image.

(9) $$\begin{array}{c} d\left(x,y\right)=f\left(x,y\right)-g\left(x,y\right). \end{array}$$

The image is preprocessed. The color image is grayed [23]. Doing so allows you to have fewer pixels, less memory for your files, and less burden on your computer when it comes to processing images. The part formula of image grayscale is as follows:

(10) $$\begin{array}{c} \text{gray}=R*0.3+G*0.59+B*0.11,\\ \text{gray}=G,\\ \text{gray}=\left(R*76+G*151+B*28\right)\gg 8,\\ \text{gray}=\frac{\left(R*30+G*0.59+B*11\right)}{100}. \end{array}$$

It means far greater than that. Four gray value formulas represent four different gray methods of images.

Binarization [24] makes the image black or white as follows:

(11) $$\begin{array}{c} g\left(x,y\right)=\left\{\begin{array}{c} 0\left(\text{gray value is less than threshold}\left(T\right)\right),\\ 255\left(\text{gray value is greater than threshold}\left(T\right)\right). \end{array}\right. \end{array}$$

In the image binarization operation, *T* represents the gray value of pixels on the image. The confirmation of *T* is mainly as follows: when the gray level is higher than the threshold pixel, it is determined to be represented by the gray level value 255; otherwise, the gray value is 0, indicating the background or exceptional object area.

**Figure 2:**
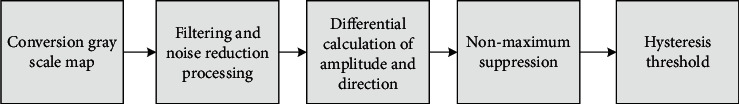
Edge detection flow of the Canny operator.

Take the Canny operator as an example, as shown in Figure 2:

Common convolution kernel templates are as follows:

(12) $$\begin{array}{c} \left[\begin{array}{ccc} \frac{1}{16} & \frac{2}{16} & \frac{1}{16}\\ \frac{2}{16} & \frac{4}{16} & \frac{2}{16}\\ \frac{1}{16} & \frac{2}{16} & \frac{1}{16} \end{array}\right]. \end{array}$$

Calculate the magnitude and direction of the gradient, and the following are the *x* direction and *y* direction:

(13) $$\begin{array}{c} S_{x}=\left[\begin{array}{ccc} -1 & 0 & 1\\ -2 & 0 & 2\\ -1 & 0 & 1 \end{array}\right],\\ S_{y}=\left[\begin{array}{ccc} -1 & -2 & -1\\ 0 & 0 & 0\\ 1 & 2 & 1 \end{array}\right]. \end{array}$$

Let the images H(i, j) and C be the gradient to be calculated.

(14) $$\begin{array}{c} H\left(i,j\right)=\left[\begin{array}{ccc} A_{0} & A_{1} & A_{2}\\ A_{3} & C & A_{5}\\ A_{6} & A_{7} & A_{8} \end{array}\right]. \end{array}$$

We can get gradients in the *x* and *y* directions, respectively, by using the following equation:

(15) $$\begin{array}{c} G_{x}=2\times A_{5}+A_{2}+A_{8}-\left(2\times A_{3}+A_{0}+A_{6}\right),\\ G_{y}=2\times A_{7}+A_{6}+A_{8}-\left(2\times A_{1}+A_{0}+A_{2}\right). \end{array}$$

At this point, the gradient amplitude and direction at point C are as follows:

(16) $$\begin{array}{c} G_{C\left(i,j\right)}=\sqrt{G_{x}^{2}-G_{y}^{2}},\\ \theta =\text{arctan}\left(\frac{G_{y}}{G_{x}}\right). \end{array}$$

### 2.4. Lip Feature Extraction Method

The lip feature extraction method [25] is shown in Table 2.

**Figure 3:**
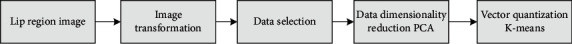
Flow chart of cascade lip feature extraction.

In this paper, we design a lip feature extraction flow chart, as shown in Figure 3, to get the final feature vector.

## 3. Design of a Collaborative System of Mouth Shape and Lyrics

### 3.1. System Development Environment

In view of the realization of the function of coordinating the subtitles in the video with the singer's mouth shape, we designed a lip-reading recognition system. The system functions are mainly divided into four functions: lip detection, feature extraction, lip-reading recognition, and automatic subtitle correction. Adjusting the appearance time of lyrics in the video makes the mouth shape coordinate with the lyrics in singing practice. The specific development environment is shown in Table 3.

### 3.2. Lip Reading Based on the HMM Model

**Figure 4:**
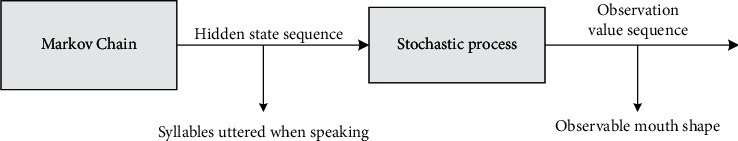
Schematic diagram of the HMM composition.

The process of the hidden Markov model is double stochastic. When people speak and sing, the staying time of each lip movement is different and it is difficult to determine. This model is consistent with the process of human lip movement, can describe the pronunciation state, and is widely used in lip reading applications. In this paper, the discrete hidden Markov model (DHMM) is adopted. As shown in Figure 4, it is the composition diagram of the HMM.

**Figure 5:**
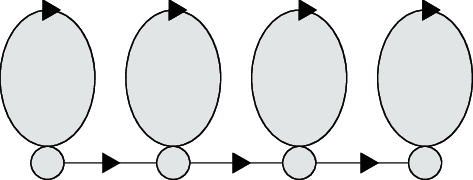
Markov chain structure.

Considering that pronunciation has strong continuity in time in the actual lip reading system, this paper chooses the topological structure of a typical Markov chain from left to right without spanning, as shown in Figure 5.

The HMM model mainly has five parameters, which can determine the model. Reasonable selection of initialization parameters can increase the recognition rate of lip reading. Where B needs to be initialized, and N chooses a coefficient of 11.

(17) $$\begin{array}{c} \lambda =\left(M,N,\pi ,A,B\right). \end{array}$$

The values of *π* and A need to meet the following conditions:

(18) $$\begin{array}{c} 0\leq \pi_{i}\leq 1,\\ \sum_{i=1}^{N}\pi_{i}=1,\\ 0\leq a_{\text{ij}}\leq 1,\\ \sum_{j=1}^{N}a_{ij}=1. \end{array}$$

**Figure 6:**
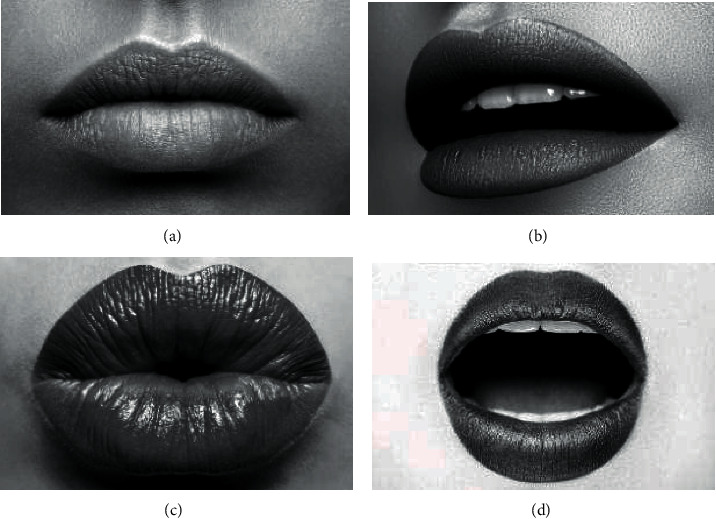
Four basic lip types of M: (a) shut up, (b) micro-opening, (c) pouting, and (d) open one's mouth.

*M* = 4, and the base mouth shape is shown in Figure 6.

### 3.3. Lip Reading Recognition and Correction

**Figure 7:**
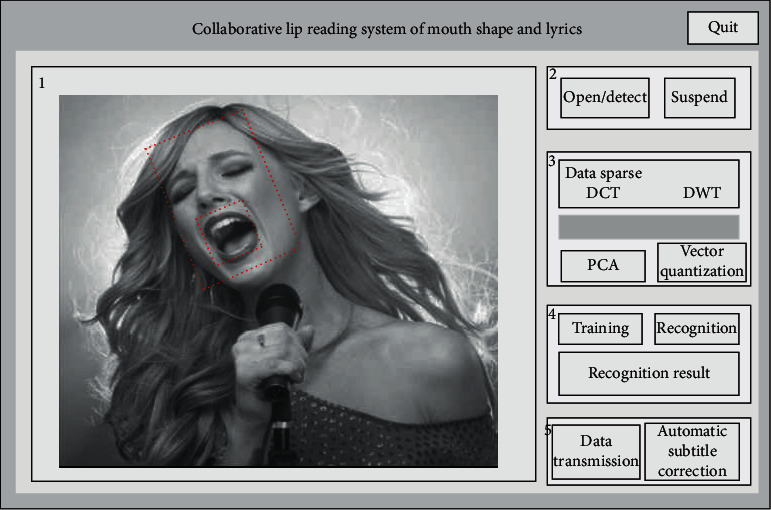
Schematic diagram of the system interface.

This research designs the interface module of the lip-reading system in which mouth shape and lyrics cooperate in singing practice. The whole interface strives to be concise and clear, and the functional distinction is clear. The first interface is designed as the placement area of each frame image of video interception, and all the specific processes of processing singing images are realized in this interface. The second interface button is designed as a lip area detection function. The third interface is the realization of the feature extraction function for the lip area. The fourth interface is the area where the image is finally recognized. After the recognition is successful, the data is automatically transmitted to the fifth interface to start the realization of automatic subtitle correction. At this time, the mouth shapes of the characters in the video will correspond to the subtitles, which successfully meets our experimental needs. The interface of the system is shown in Figure 7.

## 4. Experimental Data and Analysis

### 4.1. System Testing

#### 4.1.1. UI Testing

The purpose of UI testing is to ensure that the user interface will provide users with appropriate access or browsing functions through the functions of the test objects. The content of the test is basically perfect. Check the rationality of the system operation interface designed in this paper, and the test results are shown in Table 4.

#### 4.1.2. Performance Testing

For the experiment, the response within 2s shows that the system has the best performance; 5–10s shows that the system performance is average; if it exceeds 10s, the system performance is not good, and the system needs to be improved. The system response time is calculated as follows:

(19) $$\begin{array}{c} \text{time}=N_{1}+N_{2}+N_{3}+N_{4}+A_{1}+A_{3}+A_{2}. \end{array}$$

The network transmission time is *N*_1_+*N*_2_+*N*_3_+*N*_4_, the application server processing time is *A*_1_+*A*_3_, and the database server processing time is *A*_2_.

**Figure 8:**
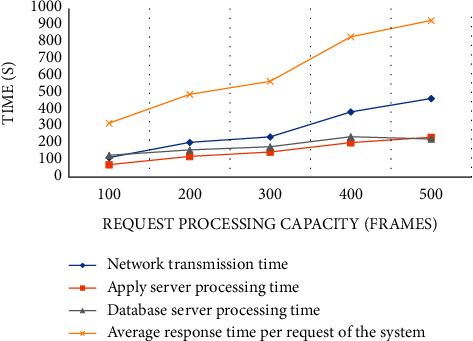
System response time.

**Figure 9:**
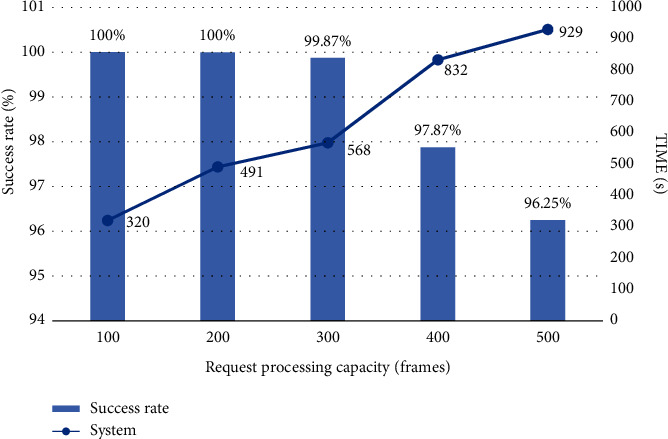
Success rate of the system response.

We select a video whose mouth shape and lyrics subtitles are not synchronized, intercept all the images for subtitle proofreading, and test the response time of the system to deal with a large number of mouth shape images at the same time. We tested the response time for 100, 200, 300, 400, and 500 frames, respectively, as shown in Figures 8 and 9.

From the specific case of system response time in Figure 8, we can find that the more image frames are requested to be processed, the longer the system response time is. The average processing time of each frame is 628 ms, and the system has good performance. From Figure 9, we can know that the success rate of system response is 98.80%, and the test results are satisfactory.

#### 4.1.3. Running Time of Each Step

**Figure 10:**
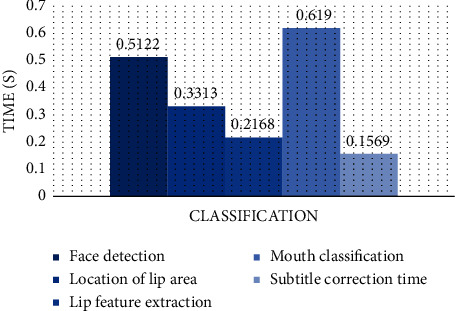
Running time statistics of each step.

The running time of each system step designed in this paper is counted, as shown in Figure 10. After calculation, we can know that the image processing speed of each step is 0.36724 s, and the most time-consuming part is lip feature extraction.

### 4.2. Lip Area Inspection Test

**Figure 11:**
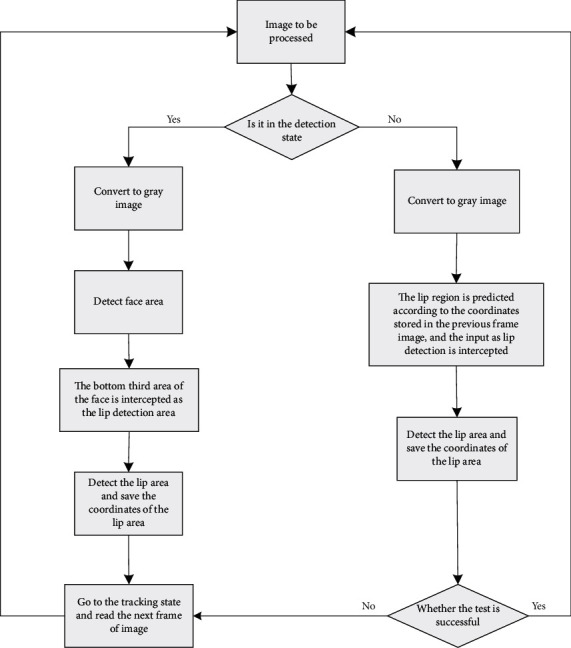
Flow chart of lip area detection.

The first step of the lip reading system is tested, and the images of characters from various angles are processed frame by frame for face recognition and lip region positioning.  Figure 11 is a flowchart of lip region detection.

**Figure 12:**
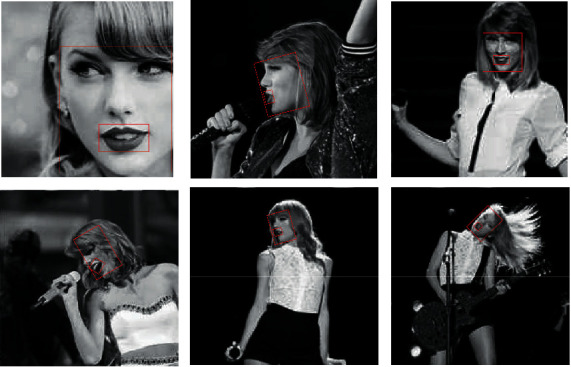
Recognition and detection renderings.

The part of the image is selected for space limitation, as shown in Figure 12. It shows face and lip recognition and detection renderings. Whether it is front or side, close-range or long-range, the system can basically accurately identify the portrait area and lip area.

### 4.3. Feature Extraction of the Lip Region

#### 4.3.1. Two Cascade Methods

**Figure 13:**
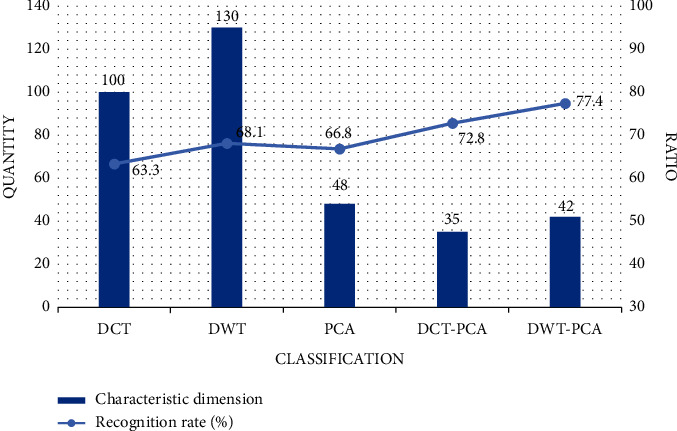
Optimal recognition rate of different features.

Using DCT-PCA and DWT-PCA, the dimension of the feature vector can be reduced by nearly 65%, and the effectiveness is greatly improved. According to the test, the recognition rate of these two methods is nearly 10% higher than that of DCT and DWT alone, as shown in Figure 13.

**Figure 14:**
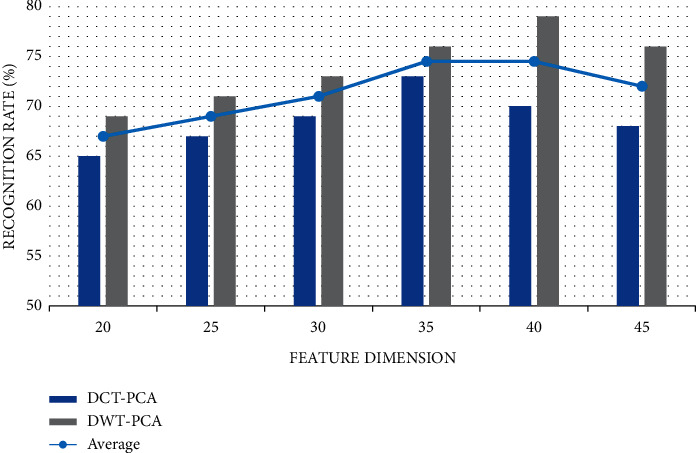
Comparison of recognition rates under different dimensions.

As shown in Figure 14, it is the change of recognition rate of two cascade methods under different feature dimensions. We can find from the figure that the higher the feature dimension, the higher the lip reading recognition rate will gradually increase within a certain range, and when it reaches a certain fixed-point peak, the recognition rate will gradually decrease slowly. This is because the number of samples in real life is limited, so when the feature dimension exceeds a certain number, it will lead to the decline of the lip reading recognition rate.

#### 4.3.2. Labial Partial Experiment

**Figure 15:**
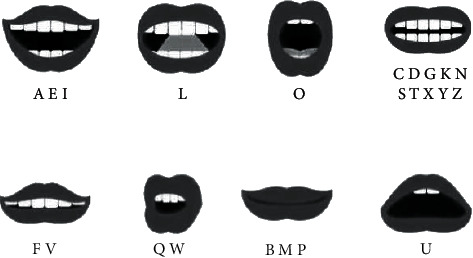
Classification according to the mouth shape of letters.

As shown in Figure 15, the mouth shapes are classified more finely.

**Figure 16:**
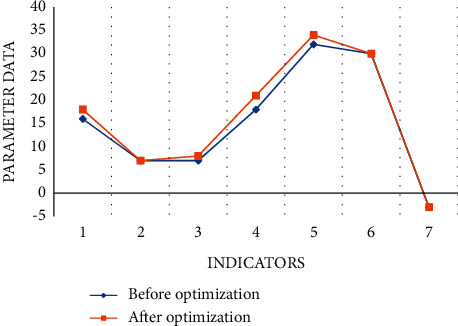
Optimization.

According to the image of human lips, lip templates are matched. Because everyone's lip shape looks different from the standard lip shape, it needs to be optimized when identifying. As shown in Figure 16, we take the O-mouth shape of an experimenter as an example. We can see that after optimization, the O-mouth shape is obviously improved, and it is easier to classify the lip shape.

### 4.4. Subtitle Correction

**Figure 17:**
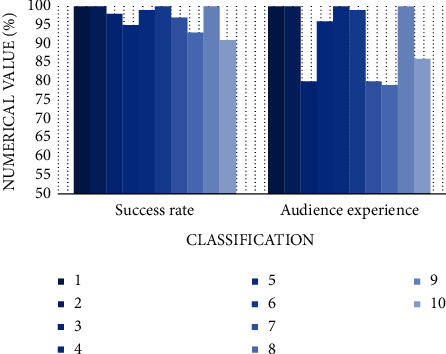
Subtitle correction diagram.

After automatic subtitle correction, the system allows the audience to experience scoring. The specific situation is shown in Figure 17. Only the correction of 10 frames of images is shown here. It can be seen that the success rate of subtitle correction is higher than 90%, with little fluctuation and good condition. However, the audience's scoring is more subjective and fluctuates more violently.

The success rate of subtitle correction judgment is higher than 90%, which is determined by objective factors. For the audience, no matter what the subtitle correction is, the audience will subjectively judge their satisfaction with the experience. This results in a large difference between the two, which is inevitable.

## 5. Conclusion

Lip-reading technology is gradually moving out of the laboratory and into real life-applications, gradually integrating into people's lives without being perceived by them as its existence and role, which is its greatest success and advantage. In this paper, image processing technology is used to accurately locate the lip area for feature extraction. According to the data of the video and audio bimodal corpus, a lip reading model based on HMM is established, so that we can recognize the mouth shape accurately and adjust the lyrics synchronously with the singers in the video, thus effectively avoiding the bad experience of the audience's unsynchronized pronunciation and character in the process of watching and singing.

The research results show the following: (1) After a UI test, the functions of various operation interfaces are reasonable. The average processing time of each image frame is 628 ms. The system has good performance, and the success rate is as high as 98.80%. The test results are satisfactory. The image processing speed of each step is 0.36724 s, and the lip feature extraction part is the most time-consuming. (2) No matter the front or side, whether the character image is close-range or long-range, the system can basically accurately identify the portrait area and lip area. (3) The recognition rate of DCT-PCA and DWT-PCA is about 10% higher than that of DCT and DWT alone, and the dimension of the feature vector is about 65% lower than that of DCT and DWT alone. When the feature dimension exceeds a certain number, the recognition rate of lip reading will decrease. (4) In order to make the mouth shape of the tester closer to the standard mouth shape in recognition, some image optimization is carried out. (5) The success rate of subtitle correction is higher than 90%, and the audience's scoring is more subjective and fluctuating.

To sum up, the whole experimental results of this study ran well. The lip-reading work has achieved good results, but due to the limitations of funds, time, and technology, there are still more details to be solved in this study. For example, there are a few different types of mouth shapes, and more pronunciation and mouth shapes need to be matched in practical application; the detection of the lip area can also separate skin color and lip color more accurately; the speed of image processing by the model can be more efficient and fast [26–28].

## Figures and Tables

**Table 1:** Main parameters of corpus.

Bimodal corpus database	
Recognition primitive	Phonemes, words, sentences
Scale	Several to dozens, even hundreds of thousands
Number of people collecting corpus	From one to dozens
Visual channel information	Color or grayscale image, storage format, frame rate
Audio channel information	Background complexity, illumination, etc.

**Table 2:** Comparison of three extraction methods.

Method	Typical algorithm	Advantages	Disadvantages
Shape-based method	Geometric feature method, snake model method, and active contour model method	Low feature dimension; it is not easy to change	The lip movement information reflected is not comprehensive; the image has clear edges
Pixel-based method	Method based on image transformation	All pixels represent visual information together, and the loss of information is relatively small	High feature dimension; image geometric transformation sensitivity
Hybrid methods	Method based on motion analysis	Combine shape-based and pixel-based features	The algorithm is complex; it is difficult to extract the ideal contour in processing

**Table 3:** Lip reading system development environment.

Design interface	MFC class library
Computer	Intel CORE i7 8th gen
Development tools	Visual studio 2020 integrated development environment
Operating system	Windows 10
Function library	OpenCV, MFC

**Table 4:** UI testing.

Serial number	Test content	Test results
1	Interface layout	Normal
2	Text display	Normal
3	Font size	Normal
4	Garbled code	None
5	Hyperlink	Normal
6	Color style	Accord with
7	Shortcut key	Normal
8	Options button	Normal
9	Text box, dialog box	Normal
10	Clarity	Clear
11	Phenomenon of jamming or flashback	None
12	Keep a certain scale	Pass

## Data Availability

The experimental data used to support the findings of this study are available from the corresponding author upon reasonable request.
